# Changes Over a Decade in Patient-Reported Outcome Measures and Minimal Clinically Important Difference Reporting in Total Joint Arthroplasty

**DOI:** 10.1016/j.artd.2023.101096

**Published:** 2023-03-06

**Authors:** Boaz Goldberg, David G. Deckey, Jens T. Verhey, Zachary K. Christopher, Mark J. Spangehl, Henry D. Clarke, Joshua S. Bingham

**Affiliations:** Department of Orthopaedic Surgery, Mayo Clinic Arizona, Phoenix, AZ, USA

**Keywords:** Total joint arthroplasty, Total knee arthroplasty, Patient-reported outcome measures (PROMs), Minimal clinically important differences (MCIDs), Total hip arthroplasty

## Abstract

**Background:**

When used appropriately, the minimal clinically important difference (MCID) provides a powerful tool for identifying meaningful improvements brought about by a given treatment, offering more clinically relevant information than frequentist statistical analysis. However, recent studies have shown inconsistent derivation methods and use of MCIDs. The goal of this study was to report the rate of patient-reported outcome measures (PROMs) and MCIDs use in the literature and assess how this rate has changed over time.

**Methods:**

All articles published in 2010 and 2020 reporting on total hip arthroplasty or total knee arthroplasty in *The Journal of Clinical Orthopaedics and Related Research*, *The Journal of Bone and Joint Surgery*, and *The Journal of Arthroplasty* were reviewed. In each reviewed article, every reported PROM and, if present, its corresponding MCID was recorded. These data were used to calculate the rate of reporting of each PROM and MCID.

**Results:**

While the total number of articles on total hip arthroplasty and total knee arthroplasty reporting PROMs increased over time, the proportion of articles reporting PROMs decreased from 49.8% (131/263) in 2010 to 35.5% (194/546) in 2020 (*P* = .011). Of these articles that report PROMs, the proportion of articles reporting any MCID increased from 2.3% (3/131) in 2010 to 16.5% (32/194) in 2020 (*P* = .002).

**Conclusions:**

The rate of reporting of MCIDs among articles relating to total hip arthroplasty and total knee arthroplasty that report PROMs has increased significantly between 2010 and 2020 but remains low. Continued emphasis on appropriate inclusion and value of MCIDs when PROMS are reported in clinical outcomes studies is needed.

## Introduction

Total hip arthroplasty (THA) and total knee arthroplasty (TKA) are 2 of the most successful and commonly performed surgeries in the United States each year [1]. The number of THAs and TKAs performed in the United States is projected to continue to grow in the coming decade [2]. The volume of these procedures has spurred tremendous research interest, with over 43,000 articles published relating to arthroplasty between 2001 and 2016 [3]. To enhance the quality of this research moving forward, there is value in understanding the methods used to conduct this research, particularly the utilization and interpretation of outcome metrics [4,5].

Ernest Codman [6] was the first to emphasize patient outcomes when he described the “end result system” in 1918. Patient-reported outcome measures (PROMs) have since become an accepted tool for quantifying the impact of hip and knee arthroplasty on patients’ lives [7]. Frequentist statistical analysis is the predominate method used to detect differences between outcome measures in current arthroplasty literature. While a significant *P* value establishes whether an observed result is not due to chance, *P* values do not describe the effect size of a treatment. An observed result may reach statistical significance but may not offer an effect size noticeable to the patient. Clinicians and researchers have consequently sought alternative methods to assess whether a treatment offers a clinically relevant effect size, such as the minimal clinically important difference (MCID). Jaeschke et al. [8] first described the concept of an MCID in 1989 as “the smallest difference in score in the domain of interest which patients perceive as beneficial and which would mandate, in the absence of troublesome side effects and excessive cost, a change in the patient’s management.” The patient acceptable symptom state is a newer metric of effect size that has been gaining popularity as a tool to evaluate the clinical impact of a procedure in regard to a patient’s symptoms. Patient acceptable symptom state, however, has had limited use thus far in the arthroplasty literature.

With the increasing frequency of PROMs reporting, the use of the MCID has also increased. While Lan et al. [9] published a study measuring the change in PROM utilization in THA research over a 15-year interval, there is a paucity of research measuring long-term trends in PROM utilization in the total joint arthroplasty literature. A previous study determined that 7.5% (129/1709) of orthopaedic clinical science articles published from 2014 to 2016 utilizing PROMs use or reference an MCID value [10,11]. However, this study was not specific to THA and TKA, and there is a lack of research regarding long-term trends in both PROM and MCID reporting in this field. Therefore, this study was undertaken to characterize changes over a decade long-term trend in PROM and MCID utilization among articles in 3, US-based, influential orthopaedic surgery journals reporting THA and TKA literature.

## Methods

No institutional review board review was required due to the nature of the study. All articles published in 3 leading, US-based, orthopaedic surgery journals reporting on THA and TKA, *The Journal of Clinical Orthopaedics and Related Research* (CORR), *The Journal of Bone and Joint Surgery* (JBJS), and *The Journal of Arthroplasty* (JOA), published in 2010 or 2020 were reviewed. Based on the title and abstract, all clinical articles related to primary THA and/or TKA were included for review. The full texts of all included articles were reviewed, and the reported PROMs and MCIDs were recorded.

An article was considered to report a PROM if it stated either the patient’s scores or a statistic immediately derived from that result (eg, the mean PROM score for a group or the proportion of a group exceeding some set score). For completeness, we included historical outcome measures, such as Harris hip score (HHS) and the Knee Society Score (KSS), which are surgeon-reported, but have historically been included as patient outcome measures. A systematic review was considered to report a PROM if it used the PROM scores from another study for meta-analysis. If an article reported a PROM, it was considered to report the associate MCID if it states the MCID’s value and/or compares the PROM scores to the MCID.

### Statistical analysis

The number of articles reporting PROMs and/or MCIDs were collected into a bespoke Excel spreadsheet (Microsoft Corp, Redmond, WA). The rate of reporting of PROMs was defined as the percentage of included articles that report any PROM or, for a specific PROM, the percentage of included articles that report the relevant PROM. The rate of reporting of MCIDs was defined as the percentage of PROM-reporting articles that report the MCID for a reported PROM or, for a specific PROM, the percentage of articles reporting the relevant PROM that also report the associated MCID. Statistical analysis utilizing JASP (JASP Team, 2022; version 0.16.1) consisted of Wilcoxon signed rank tests to compare the rates of reporting of PROMs and MCIDs between 2010 and 2020 both overall and for specific PROMs as these values were nonparametric [12]. Additional comparisons were conducted based on journal and procedure. Alpha was set to 0.05.

## Results

### Included articles

During the calendar year of 2010, 254 articles from CORR, 280 articles from JBJS, and 226 articles from JOA, for a total of 760 articles, were reviewed (Table 1). Of these articles, 75 from CORR, 43 from JBJS, and 145 from JOA, for a total of 263, reported on THA and/or TKA which were included for analysis. More specifically, 46 articles from CORR, 26 from JBJS, and 85 from JOA reported on THA (total of 157). Thirty-five from CORR, 29 from JBJS, and 83 from JOA reported on TKA (total of 147). Six from CORR, 12 from JBJS, and 23 from JOA reported on both THA and TKA.

**Table 1:** Summary of reviewed articles and rates of reporting PROMs and MCIDs.

Journal/Technique	Reviewed		Relevant/Included (% of reviewed)		Report PROMs (% of relevant)			Report MCIDs (% of report PROMs)		
	2010	2020	2010	2020	2010	2020	*P*	2010	2020	*P*
CORR										
THA or TKA	254	362	75 (29.53%)	22 (6.08%)	44 (58.67%)	5 (22.73%)	0.004	1 (2.27%)	2 (40%)	0.773
THA			46 (18.11%)	12 (3.31%)	29 (63.04%)	0 (0%)	NaN	0 (0%)	0 (N/A)	NaN
TKA			35 (13.78%)	16 (4.42%)	17 (48.57%)	5 (31.25%)	0.323	1 (5.88%)	2 (40%)	0.424
JBJS										
THA or TKA	280	201	43 (15.36%)	55 (27.36%)	16 (37.21%)	19 (34.55%)	0.546	2 (12.5%)	1 (5.26%)	0.346
THA			26 (9.29%)	37 (18.41%)	5 (19.23%)	8 (21.62%)	0.044	1 (20%)	0 (0%)	NaN
TKA			29 (10.36%)	39 (19.4%)	12 (41.38%)	13 (33.33%)	0.708	2 (16.67%)	1 (7.69%)	0.149
JOA										
THA or TKA	226	664	145 (64.16%)	469 (70.63%)	71 (48.97%)	170 (36.25%)	<0.001	0 (0%)	29 (17.06%)	NaN
THA			85 (37.61%)	302 (45.48%)	39 (45.88%)	93 (30.79%)	<0.001	0 (0%)	14 (15.05%)	NaN
TKA			83 (36.73%)	295 (44.43%)	36 (43.37%)	107 (36.27%)	<0.001	0 (0%)	19 (17.76%)	NaN
Total										
THA or TKA	760	1227	263 (34.61%)	546 (44.5%)	131 (49.81%)	194 (35.53%)	0.011	3 (2.29%)	32 (16.49%)	0.002
THA			157 (20.66%)	351 (28.61%)	73 (46.5%)	101 (28.77%)	0.004	1 (1.37%)	14 (13.86%)	0.002
TKA			147 (19.34%)	350 (28.52%)	65 (44.22%)	125 (35.71%)	<0.001	3 (4.62%)	22 (17.6%)	0.004

CORR, clinical orthopedics and related research; JBJS, journal of bone and joint surgery; JOA, journal of arthroplasty; MCID, minimal clinically important difference; THA, total hip arthroplasty; TKA, total knee arthroplasty; PROM, patient-reported outcome measure.

During the calendar year of 2020, 362 articles from CORR, 201 articles from JBJS, and 664 articles from JOA for a total of 1227 articles were reviewed (Table 1). Of these articles, 22 from CORR, 55 from JBJS, and 469 from JOA for a total of 546 reported on THA and/or TKA and were included for analysis. More specifically, 12 articles from CORR, 37 from JBJS, and 302 from JOA reported on THA (total of 351). Sixteen from CORR, 39 from JBJS, and 295 from JOA reported on TKA (total of 350). Six from CORR, 21 from JBJS, 51 from JOA reported on both THA and TKA.

### Reporting of PROMs

**Figure 1:**
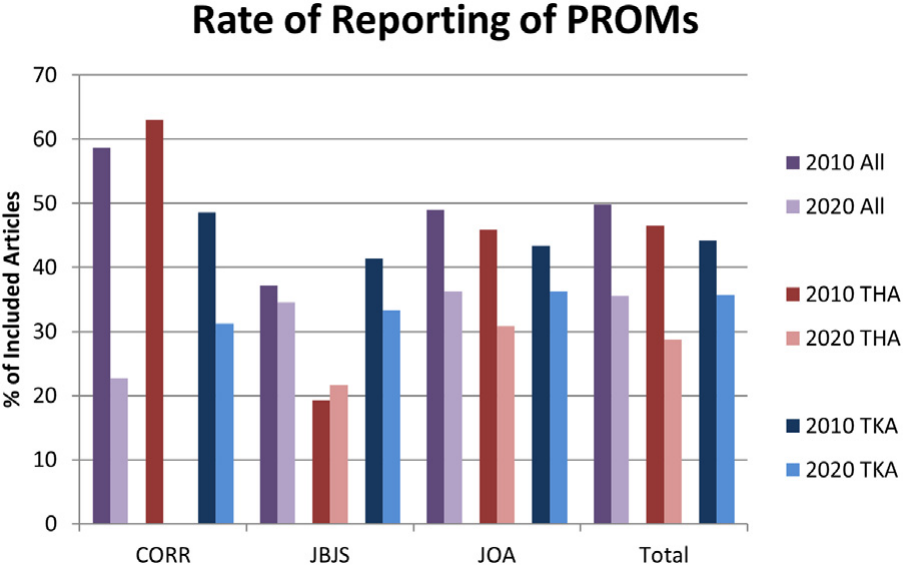
Percentage of included articles reporting at least 1 PROM by year, journal, and procedure. PROM, patient-reported outcome measure.

The number of articles reporting PROMs increased from 131 in 2010 to 194 in 2020; however, the proportion of articles reporting PROMs decreased significantly from 49.8% (131/263) in 2010 to 35.5% (194/546) in 2020 (*P* = .011) (Fig. 1). The same change persisted when analyzing THA or TKA alone. For articles related to THA, the rate of reporting of PROMs significantly decreased from 46.5% (73/157) in 2010 to 28.8% (101/351) in 2020 (*P* = .004), while for articles related to TKA, the rate of reporting of PROMs significantly decreased from 44.2% (65/147) in 2010 to 35.7% (125/350) in 2020 (*P* < .001).

**Figure 2:**
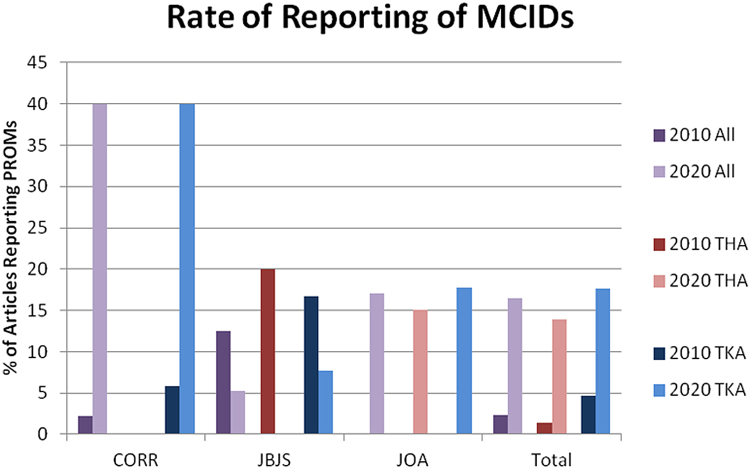
Percentage of PROM-reporting articles that also report at least 1 MCID by year, journal, and procedure. MCID, minimal clinically important difference.

**Figure 3:**
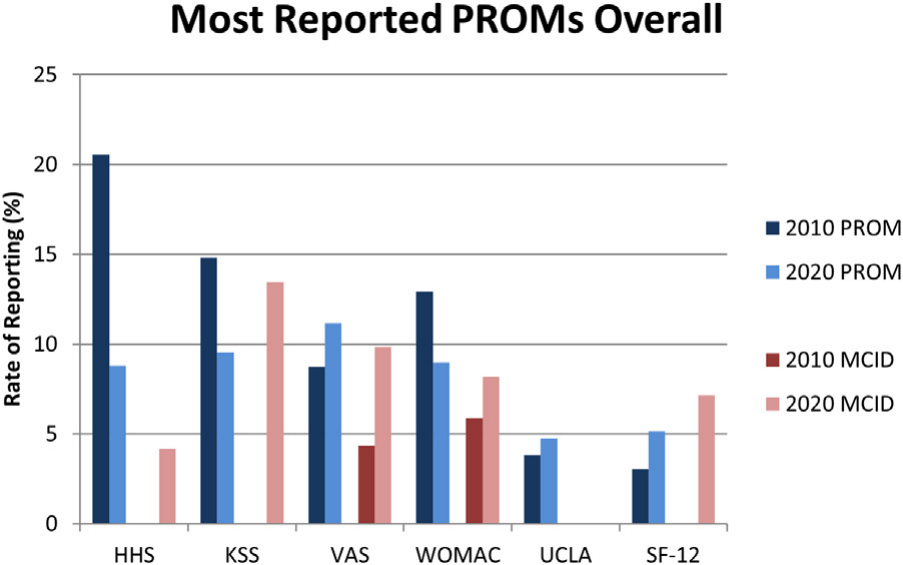
Rate of Reporting of PROMs (as the percentage of included articles that report a given PROM) and MCIDs (as the percentage of articles reporting a given PROM that also report the associated MCID) by year for the most reported PROMs as determined by the number of included articles reporting each PROM, descending left to right. MCID, minimal clinically important difference; PROM, patient-reported outcome measure.

**Figure 4:**
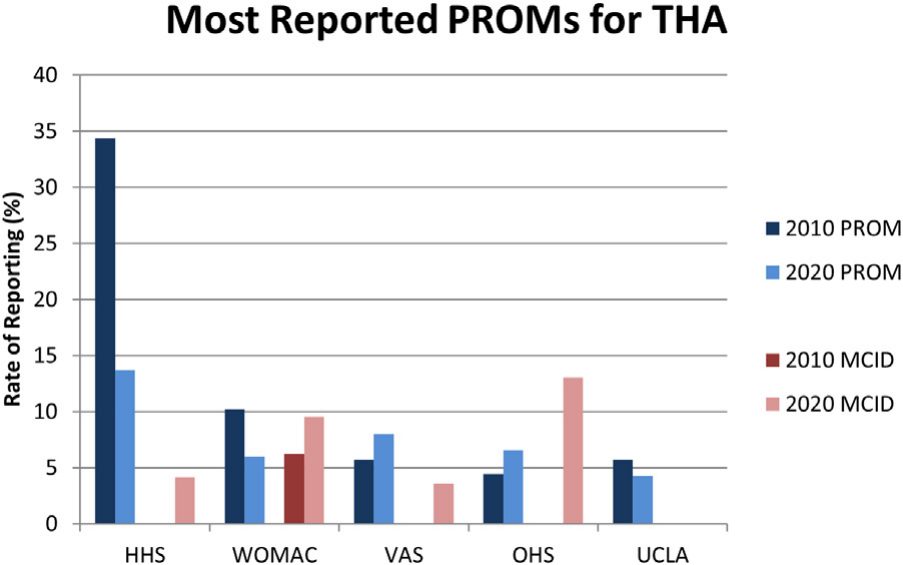
Rate of Reporting of PROMs (as the percentage of included, THA-related articles that report a given PROM) and MCIDs (as the percentage of THA-related articles reporting a given PROM that also report the associated MCID) by year for the most reported PROMs in THA research as determined by the number of included, THA-related articles reporting each PROM, descending left to right. MCID, minimal clinically important difference; PROM, patient-reported outcome measure; THA, total hip arthroplasty.

**Figure 5:**
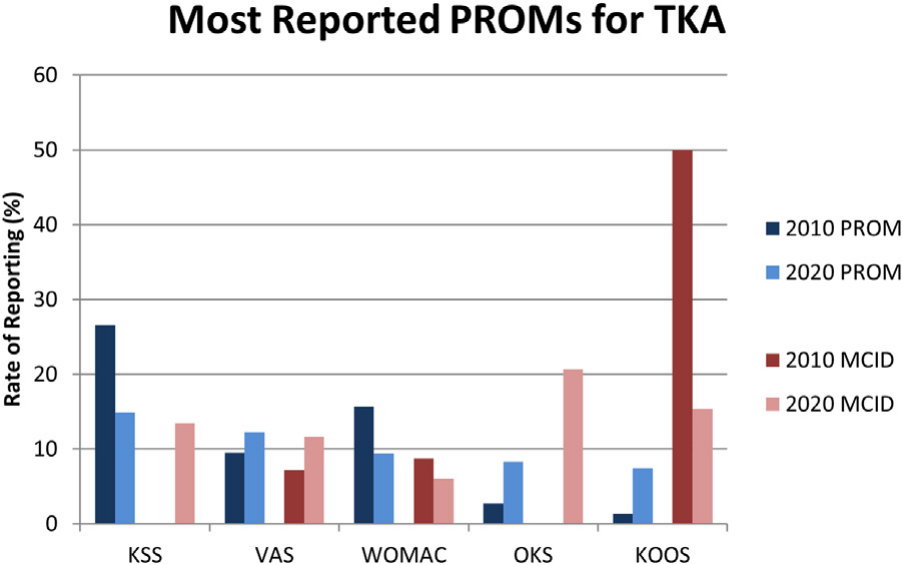
Rate of Reporting of PROMs (as the percentage of included, TKA-related articles that report a given PROM) and MCIDs (as the percentage of TKA-related articles reporting a given PROM that also report the associated MCID) by year for the most reported PROMs in TKA research as determined by the number of included, TKA-related articles reporting each PROM, descending left to right. MCID, minimal clinically important difference; PROM, patient-reported outcome measure; TKA, total knee arthroplasty.

The most reported PROMs across all included articles, in descending order by total number of articles reporting each PROM across 2010 and 2020, are the HHS(reported in 20.5% of articles in 2010; 8.8% in 2020) and the KSS (14.8% in 2010; 9.5% in 2020) (Table 2 and Fig. 3). The most reported PROMs in articles related to THA are the HHS (reported in 34.4% of THA-related articles in 2010; 13.7% in 2020), the Western Ontario and McMaster Osteoarthritis Index (10.2% in 2010; 6.0% in 2020), and the visual analog scale (VAS) (5.7% in 2010; 8.0% in 2020) (Fig. 4). Note that the visual analog scale and Western Ontario and McMaster Osteoarthritis Index are tied for the second most reported PROM among THA-related articles. The most reported PROMs in articles related to TKA are the KSS (reported in 26.5% of TKA-related articles in 2010; 14.9% in 2020), the visual analog scale (9.5% in 2010; 12.3% in 2020), and the Western Ontario and McMaster Osteoarthritis Index (15.7% in 2010; 9.4% in 2020) (Fig. 5).

**Table 2:** Rate of PROM and MCID reporting for all reported PROMs.

Patient outcome measure	2010		2020	
	Report PROM (% of included)	Report MCID (% of Report PROM)	Report PROM (% of included)	Report MCID (% of Report PROM)
Harris hip score	54 (20.53%)	0 (0%)	48 (8.79%)	2 (4.17%)
Knee Society score	39 (14.83%)	0 (0%)	52 (9.52%)	7 (13.46%)
Visual analog scale	23 (8.75%)	1 (4.35%)	61 (11.17%)	6 (9.84%)
Western Ontario and McMaster Osteoarthritis Index	34 (12.93%)	2 (5.88%)	49 (8.97%)	4 (8.16%)
Short Form-12	8 (3.04%)	0 (0%)	28 (5.13%)	2 (7.14%)
University of California Los Angeles Activity Score	10 (3.8%)	0 (0%)	26 (4.76%)	0 (0%)
Oxford Knee score	4 (1.52%)	0 (0%)	29 (5.31%)	7 (24.14%)
Oxford hip score	7 (2.66%)	0 (0%)	23 (4.21%)	3 (13.04%)
Short Form-36	16 (6.08%)	0 (0%)	13 (2.38%)	5 (38.46%)
Knee Injury and Osteoarthritis Outcome Score (KOOS)	2 (0.76%)	1 (50%)	26 (4.76%)	4 (15.38%)
EuroQol Five-Dimensions and Satisfaction (EuroQol-5D)	2 (0.76%)	0 (0%)	15 (2.75%)	1 (6.67%)
Hip Injury and Osteoarthritis Outcome Score for Joint Replacement (HOOS JR)	0 (0%)	0 (N/A)	15 (2.75%)	4 (26.67%)
Hospital for Special Surgery Score	9 (3.42%)	0 (0%)	6 (1.1%)	0 (0%)
Knee Injury and Osteoarthritis Outcome Score for Joint Replacement	0 (0%)	0 (N/A)	14 (2.56%)	2 (14.29%)
Hip Injury and Osteoarthritis Outcome Score	1 (0.38%)	0 (0%)	12 (2.2%)	3 (25%)
Forgotten Joint Score	0 (0%)	0 (N/A)	11 (2.01%)	0 (0%)
Lower Extremity Function Scale	3 (1.14%)	0 (0%)	1 (0.18%)	0 (0%)
Knee Outcome Survey	4 (1.52%)	0 (0%)	0 (0%)	0 (N/A)
Linear Analog Scale Assessment	2 (0.76%)	0 (0%)	0 (0%)	0 (N/A)
St. Michael Outcome Score	1 (0.38%)	0 (0%)	1 (0.18%)	0 (0%)
Knee Injury and Osteoarthritis Outcome Score 12	0 (0%)	0 (N/A)	1 (0.18%)	0 (0%)
Tegner Lysholm Knee Scoring Scale	0 (0%)	0 (N/A)	1 (0.18%)	0 (0%)
International Knee Documentation Committee Subjective Knee Evaluation	0 (0%)	0 (N/A)	1 (0.18%)	0 (0%)
Short Form 8	0 (0%)	0 (N/A)	1 (0.18%)	0 (0%)
Quality of Well-Being Index	1 (0.38%)	0 (0%)	0 (0%)	0 (N/A)
Paper Adaptive Test in 5 domains of Quality of Life in Arthritis Questionnaire	1 (0.38%)	0 (0%)	0 (0%)	0 (N/A)
Global Rating Scale	1 (0.38%)	0 (0%)	0 (0%)	0 (N/A)

MCID, minimal clinically important difference; PROM, patient-reported outcome measure.

### Reporting of MCIDs

Among articles that report PROMs, both the number and proportion of articles reporting MCIDs increased, changing from 2.3% (3/131) in 2010 to 16.5% (32/194) in 2020 (*P* = .002) (Fig. 2). This change persisted when analyzing articles on THA and TKA separately. For THA-related articles reporting PROMs, the proportion of articles reporting MCIDs increased from 1.4% (1/73) in 2010 to 13.9% (14/101) in 2020 (*P* = .002). For TKA-related articles reporting PROMs, the proportion of articles reporting MCIDs increased from 4.6% (3/65) in 2010 to 17.6% (22/125) in 2020 (*P* = .004).

### Differences between journals

While the rate of reporting of PROMs showed a very significant downward trend in JOA, whether analyzing THA and TKA together or separately, CORR only showed a significant decrease in PROM reporting when analyzing all articles, and JBJS showed a significant increase in PROM reporting for THA-related articles with insignificant results overall and for TKA-related articles (Fig. 1). In CORR, the rate of reporting of PROMs decreased from 58.7% (44/75) in 2010 to 22.7% (5/22) in 2020 overall (*P* = .004), decreased from 63.0% (29/46) to 0% (0/12) for THA-related articles, and decreased from 48.6% (17/35) to 31.3% (5/16) for TKA-related articles (*P* = .323). In JBJS, the rate of reporting of PROMs decreased from 37.2% (16/43) to 34.5% (19/55) overall (*P* = .546), increased from 19.2% (5/26) to 21.6% (8/37) for THA-related articles (*P* = .044), and decreased from 41.4% (12/29) to 33.3% (13/39) for TKA-related articles (*P* = .708). In JOA, the rate of reporting of PROMs decreased from 49.0% (71/145) to 36.3% (170/469) overall (*P* < .001), decreased from 45.9% (39/85) to 30.8% (93/302) for THA-related articles (*P* < .001), and decreased from 43.4% (36/83) to 36.3% (107/295) for TKA-related articles (*P* < .001).

There are no significant results when MCID reporting is broken down by journal (Fig. 2). For articles in CORR reporting PROMs, the rate of reporting of MCIDs increased from 2.3% (1/44) in 2010 to 40% (2/5) in 2020 overall (*P* = .773), remained at zero (from 0/29 to 0/0) for THA-related articles, and increased from 5.9% (1/17) to 40% (2/5) for TKA-related articles (*P* = .424). For JBJS, the rate of reporting of MCIDs decreased from 12.5% (2/16) to 5.3% (1/19) overall (*P* = .346), decreased from 20% (1/5) to 0% (0/8) for THA-related articles, and decreased from 16.7% (2/12) to 7.7% (1/13) for TKA-related articles (*P* = .149). For JOA, the rate of reporting of MCIDs increased from 0% (0/71) to 17.1% (29/170) overall, increased from 0% (0/39) to 15.1% (14/93) for THA-related articles, and increased from 0% (0/36) to 17.8% (19/107) for TKA-related articles.

## Discussion

Assessing PROMs with MCIDs is a powerful tool to quantify the effect size of differing treatment methods and determining whether these benefits are clinically meaningful. Given the well-established advantages of using PROMs and MCIDs in tandem, it is useful to understand how these metrics are being used in published research, and how this use has changed over time. Our study contributes to this understanding for the literature on total hip and knee arthroplasty. The utilization of PROMs has decreased in total hip and knee arthroplasty research; however, among articles that do report PROMs, a greater proportion are making use of their respective MCIDs.

Surprisingly, we report a decrease in the rate of reporting of PROMs in both THA and TKA. However, previous research has shown that “outcome metric reporting” has increased in articles on THA between 2005 and 2019 [9]. However, this study encompassed a broader range of metrics – for example, including “patient satisfaction” as a metric, while the current study only included specific, recognized PROMs measuring satisfaction. This may suggest that there is a trend in THA research of employing metrics other than the PROMs to evaluate patient-centric outcomes. To date, no such study reports on changes in PROMs reporting over time in TKA literature. Similarly, Vajapey et al. [13] reported 63.5% of randomized clinical trials published on THA reported “subjective hip function” assessed by PROMs. This is higher than the rate of PROM reporting in THA-related studies we calculated for 2010 (46.5%) or 2020 (29.77%) suggesting that randomized clinical trials utilize PROMs at a higher rate than research articles in general. Again, no studies have compared the rate of PROMs utilized in randomized clinical trials vs noncontrolled studies in TKA.

It is also important to note that there is variation in the quality of usage among studies that utilize PROMs. Tariq et al. [14] found that 90% of arthroplasty studies with at least 1,000 participants, 6-month follow-up, and PROM utilization had lower than an 80% follow-up rate. This can introduce significant bias and limit generalizability [[14], [15], [16], [17]]. Therefore, it is important for researchers to not only focus on utilizing appropriate outcome metrics, but also on ensuring that study design supports the effective use of these outcomes.

Although the rate of PROM utilized has decreased, MCID utilization has increased among PROM-reporting articles. We report a strong increase in the proportion of THA and TKA articles utilizing MCID. This is consistent with the limited previous research related to this subject. Copay et al. [10,11] reviewed 4462 articles from 7 major orthopaedic journals published from 2014 to 2016 and found that of the 1709 clinical sciences articles that utilized PROMs, 129 (7.5%) used or referenced the MCID. While this study did not exclusively review articles on total hip and knee arthroplasty, 61.2% of the 129 MCID-reporting studies reviewed by Copay et al. [10,11] were related to the lower extremity, suggesting that Copay’s study represents a similar population to this study. As our study found that 3.2% of articles reporting PROMs reported MCIDs in 2010 as compared to 16.5% in 2020, the findings by Copay et al [10,11] are consistent with the increase in MCID reporting suggested by this study.

While the proportion of articles utilizing MCIDs has increased, it is still important to note that the large majority of THA and TKA studies published in 2020 (83.5%) did not utilize MCIDs. This is concerning, as large studies can at times find statistically significant results from trivial effect sizes, overinflating the importance of certain results [18]. Without MCIDs, there is not an objective metric by which clinical researchers can interpret changes seen with treatment for an audience not familiar with a given outcome measure [8]. It is also worth noting that even among articles that utilize MCID values, the calculation and value of an MCID for a given metric lacks uniformity between studies [4,5,10,11]. While the increasing adoption of MCID reporting is overall a positive change, substantial variability and inconsistencies in the use and reporting of MCID values abound. No current consensus exists regarding MCID calculation and presently, the utilization of MCIDs lacks both frequency and uniformity. We recommend that journals encourage the use of the MCID whenever outcome measures are reported to estimate effect size in addition to statistical differences. In doing so, authors should utilize calculation methods of MCID consistent with previous literature or reference MCID values from study populations similar to their own.

### Limitations

This study does have limitations. The review was limited to articles published in 3, US-based, journals – CORR, JBJS, and JOA. These are 3 of the journals in arthroplasty, publishing a combined cumulatively, these journals accounted for 21.1% of research articles related to arthroplasty from 2001 to 2016 [3]. Therefore, these journals provide a greater variety of arthroplasty-related articles, but this limitation may still impact the strength of generalizations drawn from these data. Additionally, only US-based journals were queried, yet these journals publish articles from numerous international authors. However, this may limit the generalizability outside of North American literature if there is publication bias against international authors. Publication bias may also be present given the stringent criteria required to be published in these 3 journals. We also only included MCID, as we felt that at this point in time the usage of this was more prevalent, and thus more impactful to examine. While patient acceptable symptom state is a useful metric to evaluate clinical impact, at this point its use is not widespread. Future studies can look to evaluate its change in usage over time. Additionally, we included outcome measures such as the HHS and KSS, although these technically are not patient-reported outcome measures yet are in fact surgeon-completed outcome measures. We included these measures for completeness, even though they are technically administered by the surgeon.

## Conclusion

In this literature review on clinical outcomes after total hip and knee arthroplasty, the proportion of research articles utilizing PROMs decreased from 2010 to 2020. However, of the articles that did utilize PROMs, the proportion of articles utilizing the MCIDs for these PROMs increased over the same time frame. Despite this increase in the use of MCIDs, the low overall rate of inclusion in recent clinical outcome studies makes it clear that increased emphasis is needed to advocate for the inclusion of this information in future reports. We encourage editorial teams at orthopaedic journals to establish standards that define when it is appropriate to include MCIDs with PROMS in reports from clinical outcome studies.

## Conflicts of interest

The authors declare there are no conflicts of interest.

For full disclosure statements refer to https://doi.org/10.1016/j.artd.2023.101096.

## Appendix A Supplementary Data

Conflict of Interest Statement for Goldberg

Conflict of Interest Statement for Deckey

Conflict of Interest Statement for Clarke

Conflict of Interest Statement for Bingham

Conflict of Interest Statement for Verhey

Conflict of Interest Statement for Spangehl

Conflict of Interest Statement for Christopher
